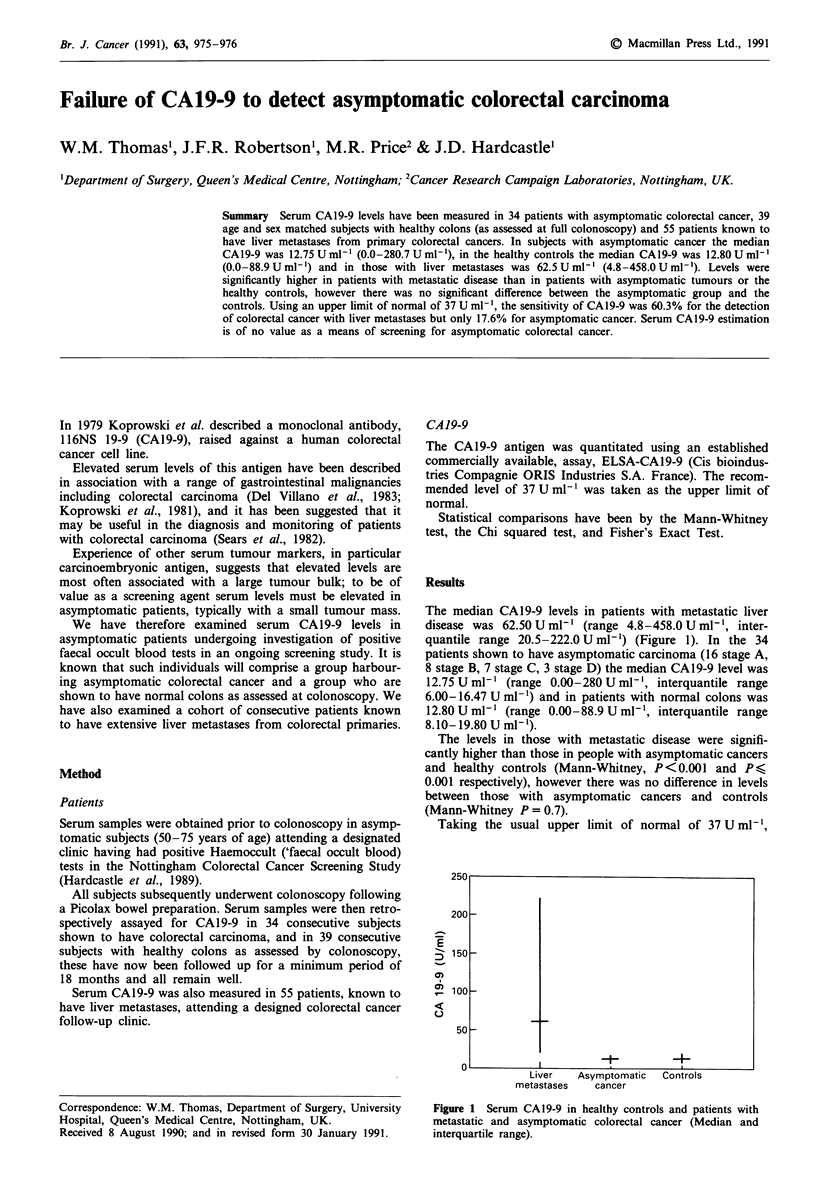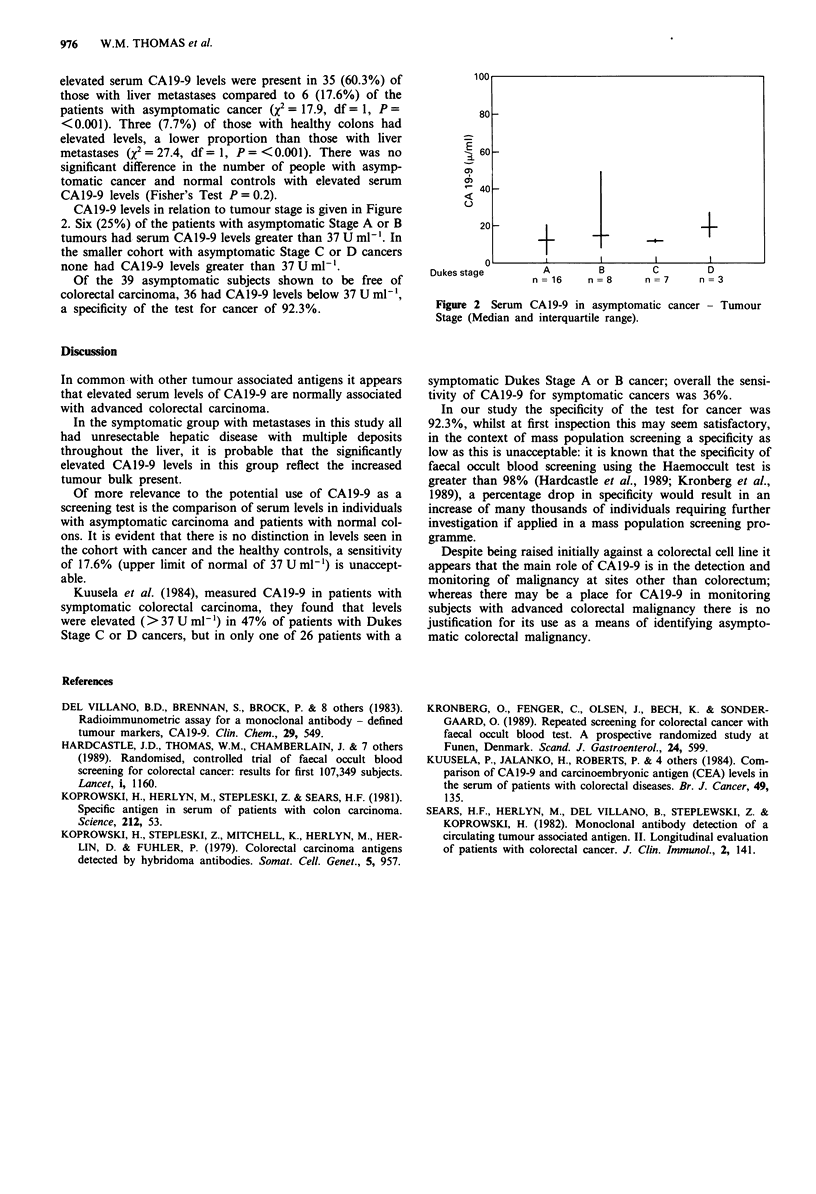# Failure of CA19-9 to detect asymptomatic colorectal carcinoma.

**DOI:** 10.1038/bjc.1991.213

**Published:** 1991-06

**Authors:** W. M. Thomas, J. F. Robertson, M. R. Price, J. D. Hardcastle

**Affiliations:** Department of Surgery, Queen's Medical Centre, Nottingham, UK.

## Abstract

Serum CA19-9 levels have been measured in 34 patients with asymptomatic colorectal cancer, 39 age and sex matched subjects with healthy colons (as assessed at full colonoscopy) and 55 patients known to have liver metastases from primary colorectal cancers. In subjects with asymptomatic cancer the median CA19-9 was 12.75 U ml-1 (0.0-280.7 U ml-1), in the healthy controls the median CA19-9 was 12.80 U ml-1 (0.0-88.9 U ml-1) and in those with liver metastases was 62.5 U ml-1 (4.8-458.0 U ml-1). Levels were significantly higher in patients with metastatic disease than in patients with asymptomatic tumours or the healthy controls, however there was no significant difference between the asymptomatic group and the controls. Using an upper limit of normal of 37 U ml-1, the sensitivity of CA19-9 was 60.3% for the detection of colorectal cancer with liver metastases but only 17.6% for asymptomatic cancer. Serum CA19-9 estimation is of no value as a means of screening for asymptomatic colorectal cancer.


Br. J. Cancer (1991), 63, 975-976 © Macmillan Press Ltd., 1991

### Failure of CA19-9 to detect asymptomatic colorectal carcinoma

W.M. Thomas[1], J.F.R. Robertson[1], M.R. Price[2] & J.D. Hardcastle[1]

[1]*Department of Surgery, Queen's Medical Centre, Nottingham;* [2]*Cancer Research Campaign Laboratories, Nottingham, UK.*

**Summary** Serum CA19-9 levels have been measured in 34 patients with asymptomatic colorectal cancer, 39 age and sex matched subjects with healthy colons (as assessed at full colonoscopy) and 55 patients known to have liver metastases from primary colorectal cancers. In subjects with asymptomatic cancer the median CA19-9 was 12.75 U $ml^{-1}$ (0.0–280.7 U $ml^{-1}$), in the healthy controls the median CA19-9 was 12.80 U $ml^{-1}$ (0.0–88.9 U $ml^{-1}$) and in those with liver metastases was 62.5 U $ml^{-1}$ (4.8–458.0 U $ml^{-1}$). Levels were significantly higher in patients with metastatic disease than in patients with asymptomatic tumours or the healthy controls, however there was no significant difference between the asymptomatic group and the controls. Using an upper limit of normal of 37 U $ml^{-1}$, the sensitivity of CA19-9 was 60.3% for the detection of colorectal cancer with liver metastases but only 17.6% for asymptomatic cancer. Serum CA19-9 estimation is of no value as a means of screening for asymptomatic colorectal cancer.

In 1979 Koprowski *et al.* described a monoclonal antibody, 116NS 19-9 (CA19-9), raised against a human colorectal cancer cell line.

Elevated serum levels of this antigen have been described in association with a range of gastrointestinal malignancies including colorectal carcinoma (Del Villano *et al.*, 1983; Koprowski *et al.*, 1981), and it has been suggested that it may be useful in the diagnosis and monitoring of patients with colorectal carcinoma (Sears *et al.*, 1982).

Experience of other serum tumour markers, in particular carcinoembryonic antigen, suggests that elevated levels are most often associated with a large tumour bulk; to be of value as a screening agent serum levels must be elevated in asymptomatic patients, typically with a small tumour mass.

We have therefore examined serum CA19-9 levels in asymptomatic patients undergoing investigation of positive faecal occult blood tests in an ongoing screening study. It is known that such individuals will comprise a group harbouring asymptomatic colorectal cancer and a group who are shown to have normal colons as assessed at colonoscopy. We have also examined a cohort of consecutive patients known to have extensive liver metastases from colorectal primaries.

### Method

*Patients*

Serum samples were obtained prior to colonoscopy in asymptomatic subjects (50–75 years of age) attending a designated clinic having had positive Haemoccult ('faecal occult blood) tests in the Nottingham Colorectal Cancer Screening Study (Hardcastle *et al.*, 1989).

All subjects subsequently underwent colonoscopy following a Picolax bowel preparation. Serum samples were then retrospectively assayed for CA19-9 in 34 consecutive subjects shown to have colorectal carcinoma, and in 39 consecutive subjects with healthy colons as assessed by colonoscopy, these have now been followed up for a minimum period of 18 months and all remain well.

Serum CA19-9 was also measured in 55 patients, known to have liver metastases, attending a designed colorectal cancer follow-up clinic.

*CA19-9*

The CA19-9 antigen was quantitated using an established commercially available, assay, ELSA-CA19-9 (Cis bioindustries Compagnie ORIS Industries S.A. France). The recommended level of 37 U $ml^{-1}$ was taken as the upper limit of normal.

Statistical comparisons have been by the Mann-Whitney test, the Chi squared test, and Fisher's Exact Test.

### Results

The median CA19-9 levels in patients with metastatic liver disease was 62.50 U $ml^{-1}$ (range 4.8–458.0 U $ml^{-1}$, interquantile range 20.5–222.0 U $ml^{-1}$) (Figure 1). In the 34 patients shown to have asymptomatic carcinoma (16 stage A, 8 stage B, 7 stage C, 3 stage D) the median CA19-9 level was 12.75 U $ml^{-1}$ (range 0.00–280 U $ml^{-1}$, interquantile range 6.00–16.47 U $ml^{-1}$) and in patients with normal colons was 12.80 U $ml^{-1}$ (range 0.00–88.9 U $ml^{-1}$, interquantile range 8.10–19.80 U $ml^{-1}$).

The levels in those with metastatic disease were significantly higher than those in people with asymptomatic cancers and healthy controls (Mann-Whitney, $P < 0.001$ and $P \leqslant 0.001$ respectively), however there was no difference in levels between those with asymptomatic cancers and controls (Mann-Whitney $P = 0.7$).

Taking the usual upper limit of normal of 37 U $ml^{-1}$,

**Figure 1** Serum CA19-9 in healthy controls and patients with metastatic and asymptomatic colorectal cancer (Median and interquartile range).

Correspondence: W.M. Thomas, Department of Surgery, University Hospital, Queen's Medical Centre, Nottingham, UK.
Received 8 August 1990; and in revised form 30 January 1991.

elevated serum CA19-9 levels were present in 35 (60.3%) of those with liver metastases compared to 6 (17.6%) of the patients with asymptomatic cancer ($\chi^2 = 17.9$, df = 1, $P = <0.001$). Three (7.7%) of those with healthy colons had elevated levels, a lower proportion than those with liver metastases ($\chi^2 = 27.4$, df = 1, $P = <0.001$). There was no significant difference in the number of people with asymptomatic cancer and normal controls with elevated serum CA19-9 levels (Fisher's Test $P = 0.2$).

CA19-9 levels in relation to tumour stage is given in Figure 2. Six (25%) of the patients with asymptomatic Stage A or B tumours had serum CA19-9 levels greater than 37 U $ml^{-1}$. In the smaller cohort with asymptomatic Stage C or D cancers none had CA19-9 levels greater than 37 U $ml^{-1}$.

Of the 39 asymptomatic subjects shown to be free of colorectal carcinoma, 36 had CA19-9 levels below 37 U $ml^{-1}$, a specificity of the test for cancer of 92.3%.

**Figure 2** Serum CA19-9 in asymptomatic cancer – Tumour Stage (Median and interquartile range).

### Discussion

In common with other tumour associated antigens it appears that elevated serum levels of CA19-9 are normally associated with advanced colorectal carcinoma.

In the symptomatic group with metastases in this study all had unresectable hepatic disease with multiple deposits throughout the liver, it is probable that the significantly elevated CA19-9 levels in this group reflect the increased tumour bulk present.

Of more relevance to the potential use of CA19-9 as a screening test is the comparison of serum levels in individuals with asymptomatic carcinoma and patients with normal colons. It is evident that there is no distinction in levels seen in the cohort with cancer and the healthy controls, a sensitivity of 17.6% (upper limit of normal of 37 U $ml^{-1}$) is unacceptable.

Kuusela *et al.* (1984), measured CA19-9 in patients with symptomatic colorectal carcinoma, they found that levels were elevated (>37 U $ml^{-1}$) in 47% of patients with Dukes Stage C or D cancers, but in only one of 26 patients with a symptomatic Dukes Stage A or B cancer; overall the sensitivity of CA19-9 for symptomatic cancers was 36%.

In our study the specificity of the test for cancer was 92.3%, whilst at first inspection this may seem satisfactory, in the context of mass population screening a specificity as low as this is unacceptable: it is known that the specificity of faecal occult blood screening using the Haemoccult test is greater than 98% (Hardcastle *et al.*, 1989; Kronberg *et al.*, 1989), a percentage drop in specificity would result in an increase of many thousands of individuals requiring further investigation if applied in a mass population screening programme.

Despite being raised initially against a colorectal cell line it appears that the main role of CA19-9 is in the detection and monitoring of malignancy at sites other than colorectum; whereas there may be a place for CA19-9 in monitoring subjects with advanced colorectal malignancy there is no justification for its use as a means of identifying asymptomatic colorectal malignancy.

### References

DEL VILLANO, B.D., BRENNAN, S., BROCK, P. & 8 others (1983). Radioimmunometric assay for a monoclonal antibody – defined tumour markers, CA19-9. *Clin. Chem.*, **29**, 549.

HARDCASTLE, J.D., THOMAS, W.M., CHAMBERLAIN, J. & 7 others (1989). Randomised, controlled trial of faecal occult blood screening for colorectal cancer: results for first 107,349 subjects. *Lancet*, **i**, 1160.

KOPROWSKI, H., HERLYN, M., STEPLESKI, Z. & SEARS, H.F. (1981). Specific antigen in serum of patients with colon carcinoma. *Science*, **212**, 53.

KOPROWSKI, H., STEPLESKI, Z., MITCHELL, K., HERLYN, M., HERLIN, D. & FUHLER, P. (1979). Colorectal carcinoma antigens detected by hybridoma antibodies. *Somat. Cell. Genet.*, **5**, 957.

KRONBERG, O., FENGER, C., OLSEN, J., BECH, K. & SONDERGAARD, O. (1989). Repeated screening for colorectal cancer with faecal occult blood test. A prospective randomized study at Funen, Denmark. *Scand. J. Gastroenterol.*, **24**, 599.

KUUSELA, P., JALANKO, H., ROBERTS, P. & 4 others (1984). Comparison of CA19-9 and carcinoembryonic antigen (CEA) levels in the serum of patients with colorectal diseases. *Br. J. Cancer*, **49**, 135.

SEARS, H.F., HERLYN, M., DEL VILLANO, B., STEPLEWSKI, Z. & KOPROWSKI, H. (1982). Monoclonal antibody detection of a circulating tumour associated antigen. II. Longitudinal evaluation of patients with colorectal cancer. *J. Clin. Immunol.*, **2**, 141.